# Supplementary material for: Metagenomic binning with assembly graph embeddings
Source: Bioinformatics. 2022 Aug 16;38(19):4481–7. doi: 10.1093/bioinformatics/btac557 (PMC9525014; doi:10.1093/bioinformatics/btac557)
Supplement: btac557_supplementary_data [file btac557_supplementary_data.zip › amber outputs.zip!/amber outputs/strong100/semibin_results/genome/semibintrain/heatmap_SAMPLEID.pdf]

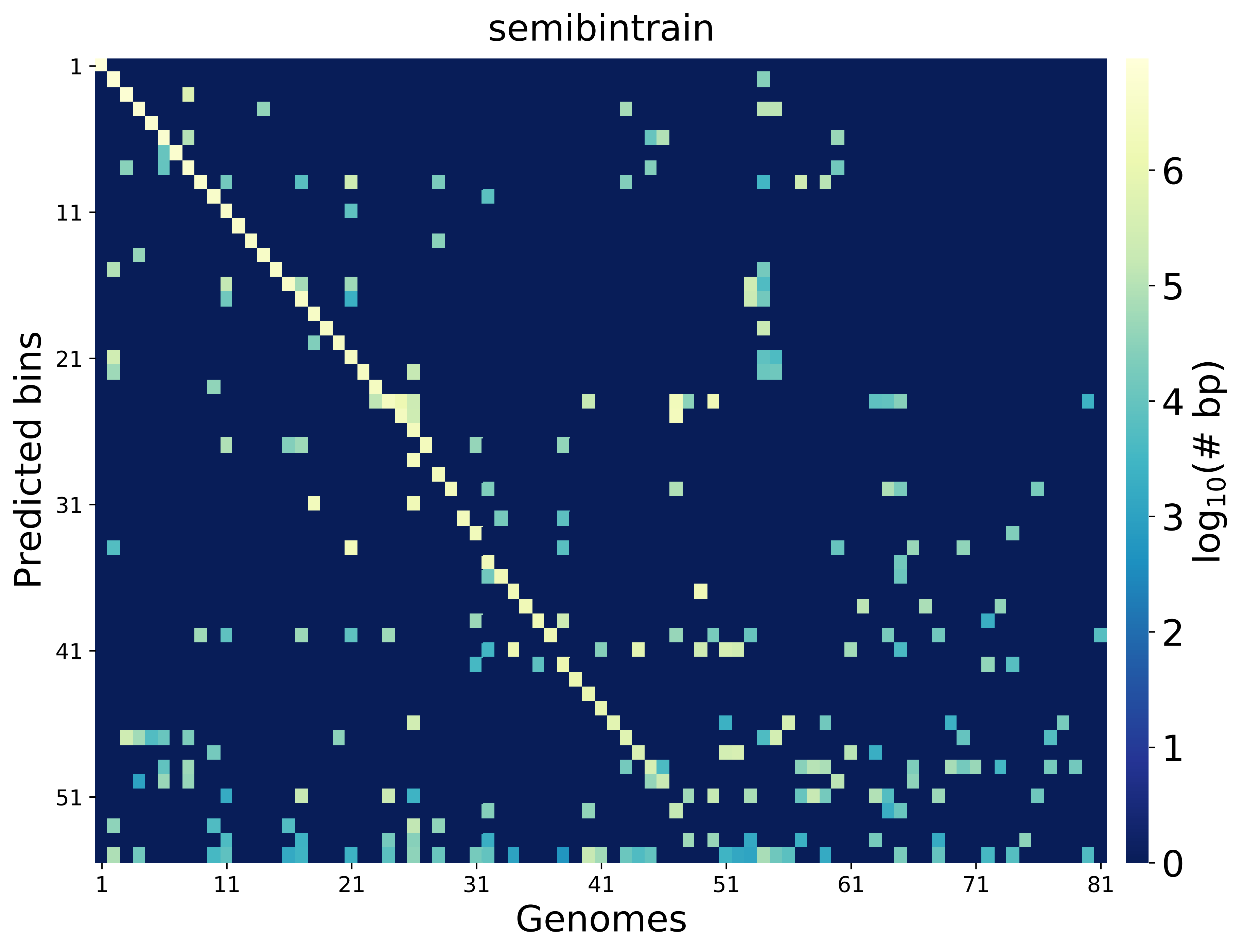

semibintrain
Predicted bins
Genomes
log10(# bp)
1
11
21
31
41
51
61
71
81
0
1
2
3
4
5
6
